# The District Nurse in the Outer Hebrides

**Published:** 1920-04-10

**Authors:** 


### The District Nurse in the Outer Hebrides.

Perhaps nowhere is the work of the district nurse more indispensable than in the Outer Hebrides. In a typical parish of the island of Lewis, which is over twenty-four miles in length, there are three nurses and only one doctor. A tourist, motoring along the dreary road, would not guess at one-tenth of the population; yet over 7,000 souls live on the land, mainly in "black huts," grouped together in "townships," and approached by rough roads or cart tracks. These huts stand back in a waste of mud, amid haystacks and piles of neatly cut peats. They are thatched and, for the most part, have no chimneys and only minute windows. The doors are low; to the left live the cattle, the family to the right. On the mud floor of the outer room burns a peat fire, over which a cauldron is suspended from the roof; there are seats all round. In the inner chamber the family, however numerous, sleeps in box beds.

Often the old people "have no English," so the nurse must speak Gaelic and interpret for the "Tassenach" doctor. It is hard for her to find a white house in which to live. The distances are so great that she must have a bicycle, yet owing to the gales from the Atlantic and the mud on the roads she has to lead it half the time. In winter, when the sun is up for barely five hours, much of her work has to be done in the dark. She cannot count on early consultations with the doctor, as he does not receive his telegrams till about eleven, and cannot set out on his round before then. Both roads and motors are notoriously bad in Lewis, and the nurse has to be prepared to meet emergencies alone.

It almost goes without saying that pulmonary tuberculosis is the chief scourge of the islanders, but besides this there are many cases of pneumonia, and often the nurse has her hands full with a peculiar type of epidemic enteritis with raised temperature, a fairly rapid pulse, abdominal distention, accompanied by pain and tenderness, with some diarrhœa, which spreads with alarming rapidity from hut to hut. Crofters often stand at their doors for hours watching for the advent of doctor or nurse, as they lack the energy to go to the Post Office and telegraph for aid. Hospital accommodation on the island is limited, and acute cases are confronted with the prospect of a stormy voyage before they can reach one of our large infirmaries. Thus the mud hut has often to be transformed into a theatre, and the nurse has to assist the doctor in performing emergency operations.

The difficulties of midwifery are enhanced by the fact that the beds are so inconvenient that the women have to be confined on the floor. Moreover, their fear of anæsthesia is such that, even when the use of forceps is necessary, they prefer to have them applied without chloroform.

As a rule the men, who have mostly been at sea, are more intelligent, and make better nurses than the women, but though they lack initiative no more grateful and obedient patients could be found anywhere. Now that Lord Leverhulme has bought, and is developing the Lewis, there is talk of change and improvement in every direction, and better housing for the nurses is one of the schemes mooted. Let us hope that before long they may have comfortable homes, for many years must elapse before the "black huts" disappear, and until these primitive dwellings become picturesque memories the lot of the district nurse in Lewis must be a hard one.

